# The genome sequence of the tree wasp, *Dolichovespula sylvestris *Scopoli, 1763

**DOI:** 10.12688/wellcomeopenres.17783.1

**Published:** 2022-03-28

**Authors:** Steven Falk, Darwin Tree of Life Barcoding collective, Wellcome Sanger Institute Tree of Life programme, Wellcome Sanger Institute Scientific Operations: DNA Pipelines collective, Tree of Life Core Informatics collective, Gavin R. Broad, Darwin Tree of Life Consortium

**Affiliations:** 1 Independent Researcher, Kenilworth, Warwickshire, UK; 2 Department of Life Sciences, Natural History Museum, London, UK

**Keywords:** Dolichovespula sylvestris, tree wasp, genome sequence, chromosomal, Hymenoptera

## Abstract

We present a genome assembly from an individual male *Dolichovespula sylvestris *(the tree wasp; Arthropoda; Insecta; Hymenoptera; Vespidae). The genome sequence is 233 megabases in span. The majority of the assembly (95.56%) is scaffolded into 26 chromosomal pseudomolecules. The mitochondrial genome was also assembled and is 21.3 kilobases in length.

## Species taxonomy

Eukaryota; Metazoa; Ecdysozoa; Arthropoda; Hexapoda; Insecta; Pterygota; Neoptera; Endopterygota; Hymenoptera; Apocrita; Aculeata; Vespoidea; Vespidae; Vespinae; Dolichovespula; *Dolichovespula sylvestris* Scopoli, 1763 (NCBI:txid85444).

## Background

The tree wasp, *Dolichovespula sylvestris*, is a eusocial vespine wasp that builds relatively small paper nests with a grey envelope in concealed locations, often in cavities or underground, belying its name, although it will build in protected sites in trees ( Spredbery, 1973). Nests typically have three or four layers of comb and have a maximum of a little over 200 workers at their peak ( Archer, 2012). The colony cycle is short, typically from late May to the end of September, with males produced from late July ( Edwards, 1980). Males form mating aggregations ( Edwards, 1980), but queens typically mate with one or very few males ( Foster & Ratnieks, 2001) meaning that most workers in a nest share the same parentage and are more closely related to their siblings than to the offspring of a fellow worker; worker reproduction is responsible for 50% of male eggs laid in a nest, but almost all of these are destroyed by fellow workers (‘worker policing’) or by the queen ( Wenseleers, *et al.*, 2005). As with other social wasps in temperate climates, colonies are annual and only the mated queens over-winter, workers and males dying in the autumn. The new nest is constructed by the queen and the cycle begins again.

*Dolichovespula sylvestris* has a wide range throughout the Palaearctic, reaching 66°N ( Archer, 2012). It is a widespread species in Britain and Ireland, including on islands, but is thought to be declining ( Edwards & Telfer, 2002). Nests can be usurped throughout much of its range by the socially parasitic *Dolichovespula omissa*, but this species has not been found in Britain yet. As with other vespines, a wide variety of food is taken, which is mostly made up of insects but can include carrion. Adult wasps are frequent flower visitors and are considered to be important pollinators of common figwort ( *Scrophularia nodosa*) ( Proctor *et al.*, 1996).

The evolution of social behaviour in the Hymenoptera is a huge area of research. Until recently, there had been no Vespinae genomes available, so the publication of genomes of species of *Vespa* ( Crowley *et al.*, 2022), *Vespula* ( Crowley *et al.*, 2021) and now *Dolichovespula* complement the existing genomes of Polistinae ( Patalano *et al.*, 2015; Standage *et al.*, 2016), the other vespid subfamily with eusocial societies.

## Genome sequence report

**Figure 1.**
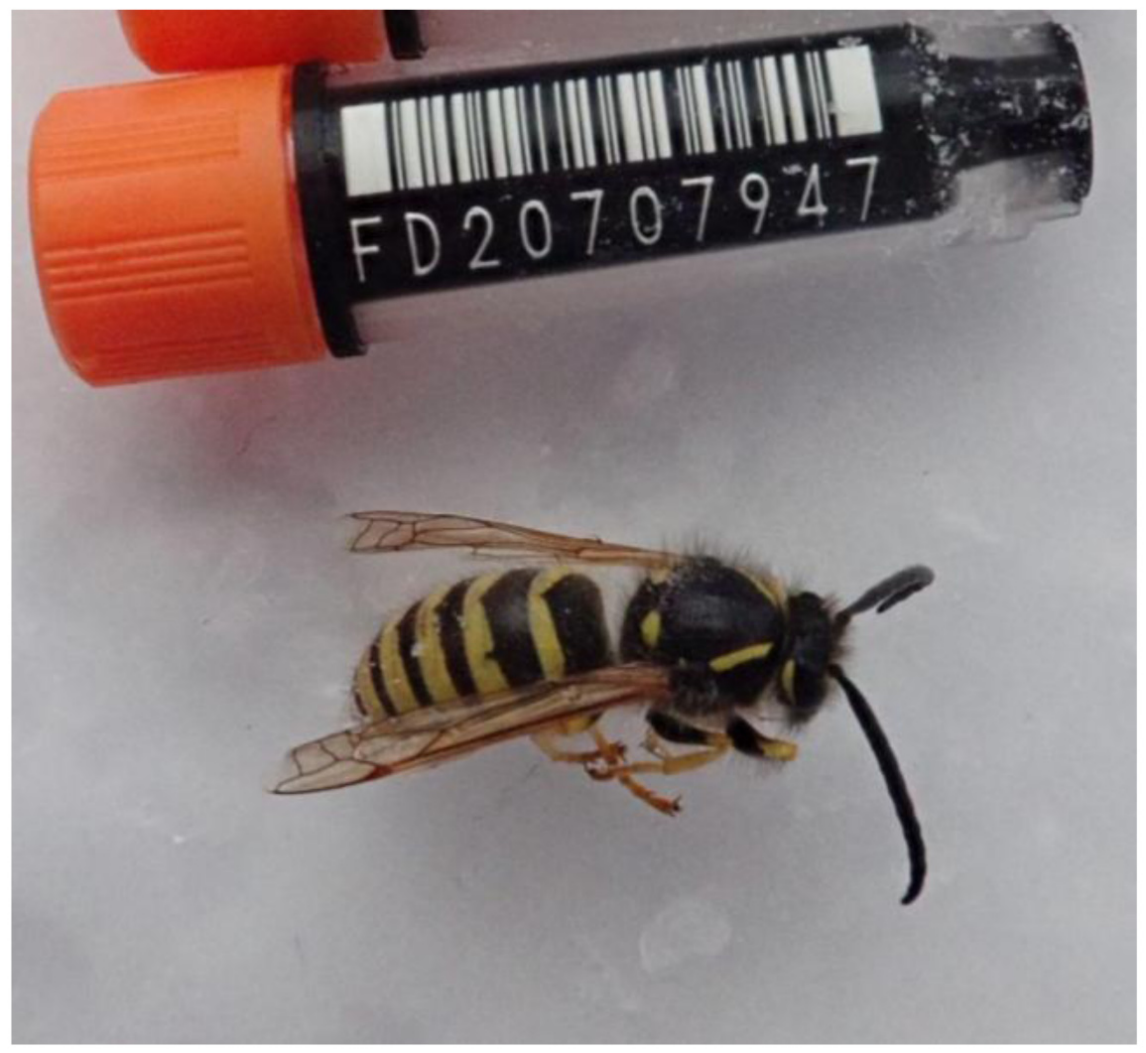
Image of the iyDolSylv1 specimen taken prior to preservation and processing.

The genome was sequenced from a single male *D. sylvestris* ( Figure 1) collected from Wytham Woods, Oxfordshire (biological vice-county: Berkshire), UK (latitude 51.770, longitude -1.331). A total of 27-fold coverage in Pacific Biosciences single-molecule long reads and 175-fold coverage in 10X Genomics read clouds were generated. Primary assembly contigs were scaffolded with chromosome conformation Hi-C data. Manual assembly curation corrected 158 missing/misjoins, increasing the assembly size by 3.47%, the scaffold number by 40.00% and the scaffold N50 by 70.68%.

**Figure 2.**
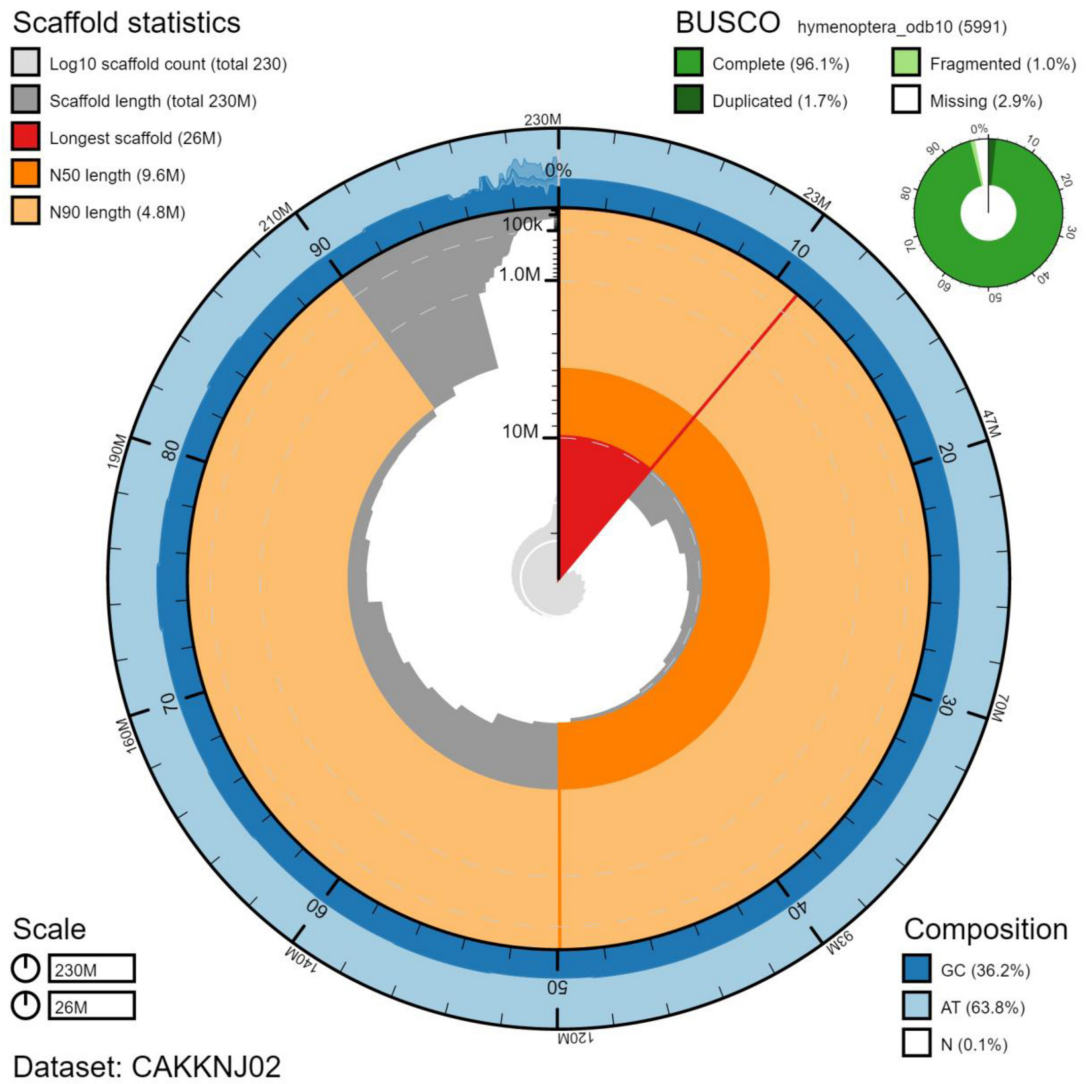
Genome assembly of *Dolichovespula sylvestris*, iyDolSylv1.2: metrics. The BlobToolKitSnailplot shows N50 metrics and BUSCO gene completeness. The main plot is divided into 1,000 size-ordered bins around the circumference with each bin representing 0.1% of the 232,601,616 bp assembly. The distribution of scaffold lengths is shown in dark grey with the plot radius scaled to the longest scaffold present in the assembly (25,955,835 bp, shown in red). Orange and pale-orange arcs show the N50 and N90 scaffold lengths (9,596,145 and 4,826,817 bp), respectively. The pale grey spiral shows the cumulative scaffold count on a log scale with white scale lines showing successive orders of magnitude. The blue and pale-blue area around the outside of the plot shows the distribution of GC, AT and N percentages in the same bins as the inner plot. A summary of complete, fragmented, duplicated and missing BUSCO genes in the hymenoptera_odb10 set is shown in the top right. An interactive version of this figure is available at https://blobtoolkit.genomehubs.org/view/iyDolSylv1.2/dataset/CAKKNJ02/snail.

The final assembly has a total length of 233 Mb in 224 sequence scaffolds with a scaffold N50 of 9.6 Mb ( Table 1). Of the assembly sequence, 95.56% was assigned to 26 chromosomal-level scaffolds (numbered by sequence length) ( Figure 2– Figure 5; Table 2). The assembly has a BUSCO v5.2.2 ( Manni *et al.*, 2021) completeness of 96.1% (single 94.4%, duplicated 1.7%) using the hymenoptera_odb10 reference set (n=5991).

**Table 1.** Genome data for *Dolichovespula sylvestris*, iyDolSylv1.2.

*Project accession data*	
Assembly identifier	iyDolSylv1.2
Species	*Dolichovespula sylvestris*
Specimen	iyDolSylv1
NCBI taxonomy ID	NCBI:txid85444
BioProject	PRJEB46852
BioSample ID	SAMEA7746475
Isolate information	Male: thorax (genome assembly), head (Hi-C)
*Raw data accessions*	
PacificBiosciences SEQUEL II	ERR6808023
10X Genomics Illumina	ERR6688641-ERR6688644
Hi-C Illumina	ERR6688640
*Genome assembly*	
Assembly accession	GCA_918808275.2
Span (Mb)	233
Number of contigs	547
Contig N50 length (Mb)	1.2
Number of scaffolds	224
Scaffold N50 length (Mb)	9.6
Longest scaffold (Mb)	26.0
BUSCO * genome score	C:96.1%[S:94.4%,D:1.7%],F:1.0%, M:2.9%,n:5991

*BUSCO scores based on the hymenoptera_odb10 BUSCO set using v5.2.2. C= complete [S= single copy, D=duplicated], F=fragmented, M=missing, n=number of orthologues in comparison. A full set of BUSCO scores is available at https://blobtoolkit.genomehubs.org/view/iyDolSylv1.2/dataset/CAKKNJ02/busco.

**Figure 3.**
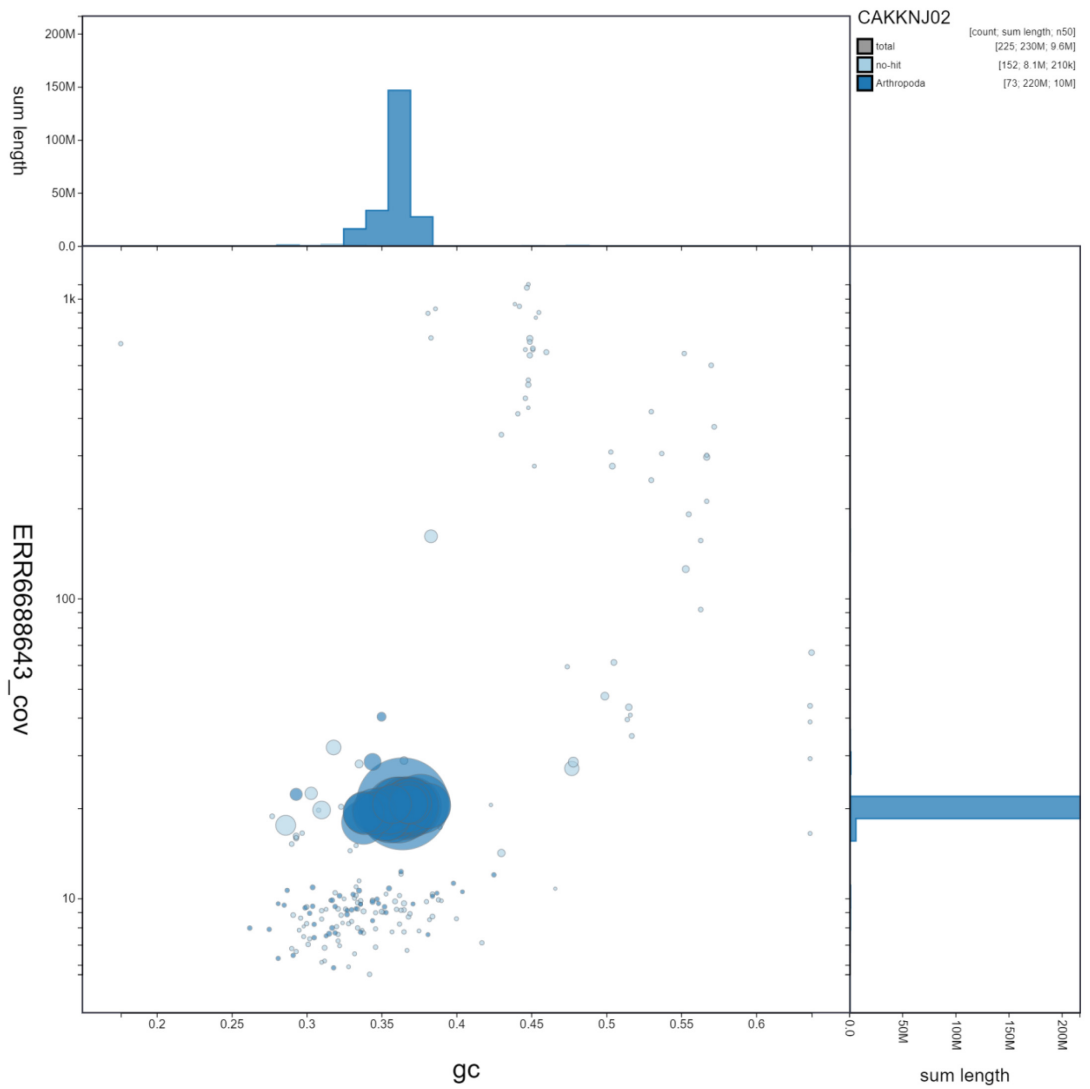
Genome assembly of *Dolichovespula sylvestris*, iyDolSylv1.2: GC coverage. BlobToolKit GC-coverage plot. Scaffolds are coloured by phylum. Circles are sized in proportion to scaffold length. Histograms show the distribution of scaffold length sum along each axis. An interactive version of this figure is available at https://blobtoolkit.genomehubs.org/view/iyDolSylv1.2/dataset/CAKKNJ02/blob.

**Figure 4.**
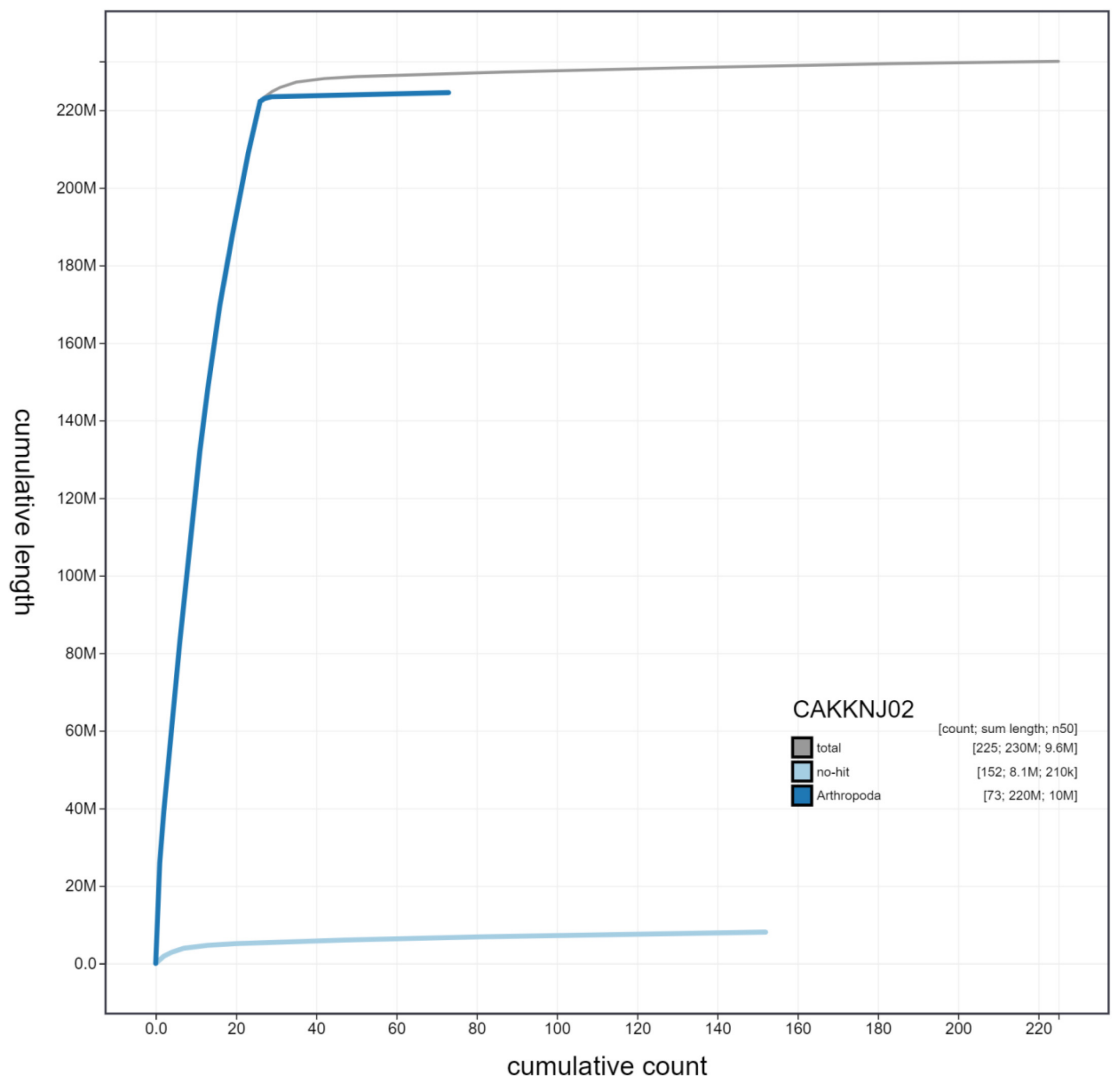
Genome assembly of *Dolichovespula sylvestris*, iyDolSylv1.2: cumulative sequence. BlobToolKit cumulative sequence plot. The grey line shows cumulative length for all scaffolds. Coloured lines show cumulative lengths of scaffolds assigned to each phylum using the buscogenestaxrule. An interactive version of this figure is available at https://blobtoolkit.genomehubs.org/view/iyDolSylv1.2/dataset/CAKKNJ02/cumulative.

**Figure 5.**
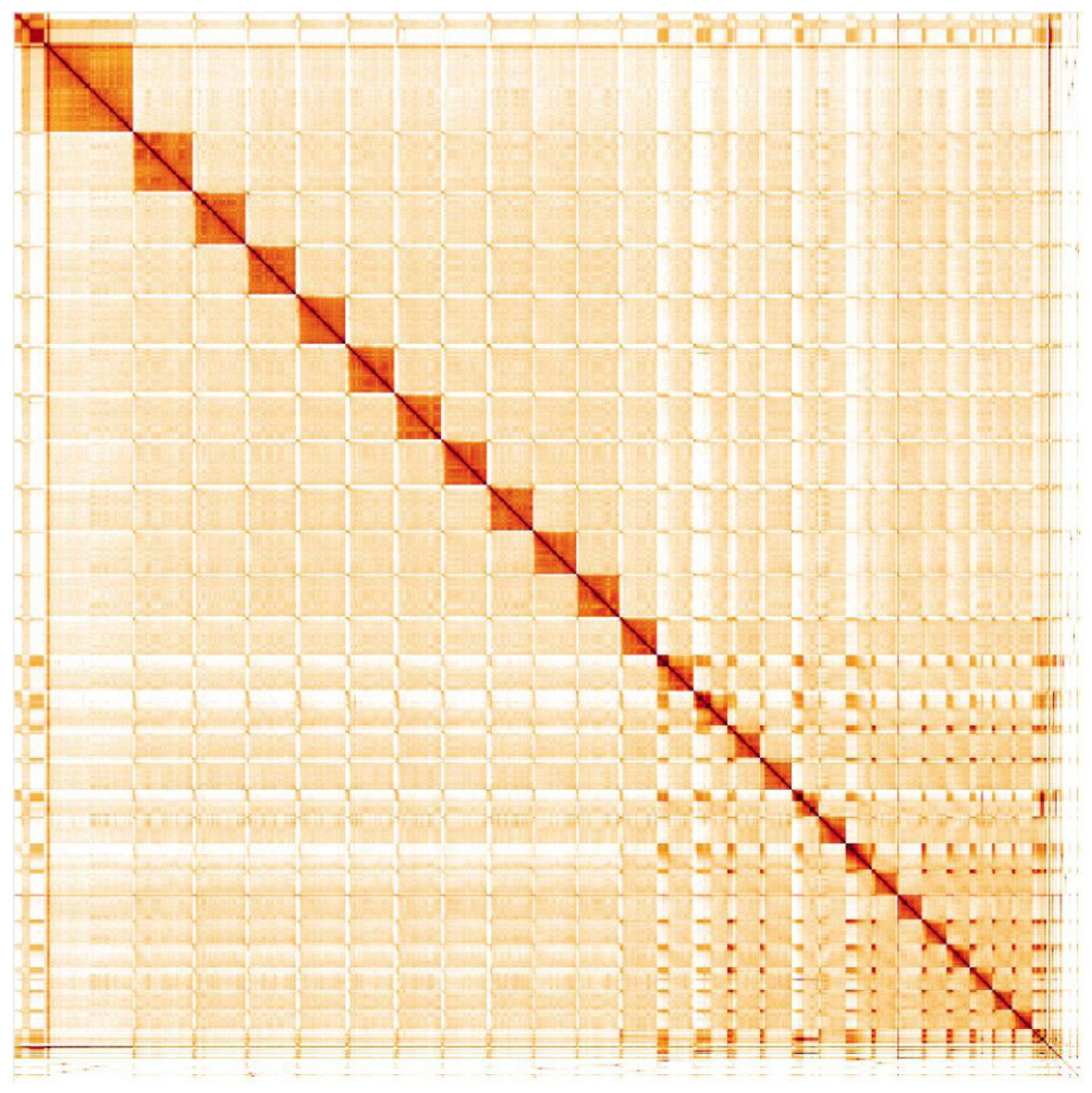
Genome assembly of *Dolichovespula sylvestris*, iyDolSylv1.2: Hi-C contact map. Hi-C contact map of the iyDolSylv1.2assembly, visualised in HiGlass. Chromosomes are shown in size order from left to right and top to bottom. An interactive version of this map is available here.

**Table 2.** Chromosomal pseudomolecules in the genome assembly of *Dolichovespula sylvestris*, iyDolSylv1.2.

INSDC accession	Chromosome	Size (Mb)	GC%
OU964961.1	1	25.96	36.4
OU964962.1	2	13.13	36.1
OU964963.1	3	11.49	36.2
OU964964.1	4	11.01	36.7
OU964965.1	5	10.78	37.0
OU964966.1	6	10.61	37.6
OU964967.1	7	10.22	36.6
OU964968.1	8	9.99	36.7
OU964969.1	9	9.98	36.9
OU964970.1	10	9.60	35.6
OU964971.1	11	9.35	36.9
OU964972.1	12	8.38	36.0
OU964973.1	13	7.81	35.3
OU964974.1	14	7.47	36.7
OU964975.1	15	7.06	35.1
OU964976.1	16	6.94	35.3
OU964977.1	17	6.00	38.1
OU964978.1	18	5.96	35.6
OU964979.1	19	5.62	33.8
OU964980.1	20	5.46	33.9
OU964981.1	21	5.39	36.9
OU964982.1	22	5.35	34.5
OU964983.1	23	5.25	34.5
OU964984.1	24	4.83	33.8
OU964985.1	25	4.33	36.8
OU964986.1	26	4.31	35.7
OU964987.1	MT	0.02	17.8
-	Unplaced	10.30	36.4

## Methods

### Sample acquisition and DNA extraction

A single male *D. sylvestris* (iyDolSylv1) was collected from Wytham Woods, Oxfordshire (biological vice-county: Berkshire), UK (latitude 51.770, longitude -1.331) by Steven Falk, independent researcher, from woodland using a net. The sample was identified by the same individual and snap-frozen on dry ice.

DNA was extracted at the Tree of Life laboratory, Wellcome Sanger Institute. The iyDolSylv1 sample was weighed and dissected on dry ice with tissue set aside for Hi-C sequencing. Thorax tissue was cryogenically disrupted to a fine powder using a Covaris cryoPREP Automated Dry Pulveriser, receiving multiple impacts. Fragment size analysis of 0.01–0.5 ng of DNA was then performed using an Agilent FemtoPulse. High molecular weight (HMW) DNA was extracted using the Qiagen Plant MagAttract HMW DNA extraction kit. Low molecular weight DNA was removed from a 200-ng aliquot of extracted DNA using 0.8X AMpure XP purification kit prior to 10X Chromium sequencing; a minimum of 50 ng DNA was submitted for 10X sequencing. HMW DNA was sheared into an average fragment size between 12–20 kb in a Megaruptor 3 system with speed setting 30. Sheared DNA was purified by solid-phase reversible immobilisation using AMPure PB beads with a 1.8X ratio of beads to sample to remove the shorter fragments and concentrate the DNA sample. The concentration of the sheared and purified DNA was assessed using a Nanodrop spectrophotometer and Qubit Fluorometer and Qubit dsDNA High Sensitivity Assay kit. Fragment size distribution was evaluated by running the sample on the FemtoPulse system.

### Sequencing

Pacific Biosciences HiFi circular consensus and 10X Genomics Chromium read cloud sequencing libraries were constructed according to the manufacturers’ instructions. Sequencing was performed by the Scientific Operations core at the Wellcome Sanger Institute on Pacific Biosciences SEQUEL II (HiFi) and Illumina NovaSeq 6000 (10X) instruments. Hi-C data were generated from head tissue of iyDolSylv1 using the Arima v2 kit and sequenced on NovaSeq 6000.

### Genome assembly

Assembly was carried out with Hifiasm ( Cheng *et al.*, 2021). Haplotypic duplication was identified and removed with purge_dups( Guan *et al.*, 2020). One round of polishing was performed by aligning 10X Genomics read data to the assembly with longranger align, calling variants with freebayes( Garrison & Marth, 2012). The assembly was then scaffolded with Hi-C data ( Rao *et al.*, 2014) using SALSA2 ( Ghurye *et al.*, 2019). The assembly was checked for contamination as described previously ( Howe *et al.*, 2021). Manual curation was performed using HiGlass ( Kerpedjiev *et al.*, 2018) and Pretext. The mitochondrial genome was assembled using MitoHiFi ( Uliano-Silva *et al.*, 2021), which performed annotation using MitoFinder ( Allio *et al.*, 2020). The genome was analysed and BUSCO scores generated within the BlobToolKit environment ( Challis *et al.*, 2020). Table 3 contains a list of all software tool versions used, where appropriate.

**Table 3.** Software tools used.

Software tool	Version	Source
Hifiasm	0.15.1-r334	Cheng *et al.*, 2021
purge_dups	1.2.3	Guan *et al.*, 2020
SALSA2	2.2	Ghurye *et al.*, 2019
longranger align	2.2.2	https://support.10xgenomics.com/ genome-exome/software/pipelines/ latest/advanced/other-pipelines
freebayes	v1.3.1-17-gaa2ace8	Garrison & Marth, 2012
MitoHiFi	2.0	Uliano-Silva *et al.*, 2021
HiGlass	1.11.6	Kerpedjiev *et al.*, 2018
PretextView	0.1.x	https://github.com/wtsi-hpag/ PretextView
BlobToolKit	3.0.5	Challis *et al.*, 2020

### Ethics/compliance issues

The materials that have contributed to this genome note have been supplied by a Darwin Tree of Life Partner. The submission of materials by a Darwin Tree of Life Partner is subject to the Darwin Tree of Life Project Sampling Code of Practice. By agreeing with and signing up to the Sampling Code of Practice, the Darwin Tree of Life Partner agrees they will meet the legal and ethical requirements and standards set out within this document in respect of all samples acquired for, and supplied to, the Darwin Tree of Life Project. Each transfer of samples is further undertaken according to a Research Collaboration Agreement or Material Transfer Agreement entered into by the Darwin Tree of Life Partner, Genome Research Limited (operating as the Wellcome Sanger Institute), and in some circumstances other Darwin Tree of Life collaborators.

## Data availability

### Underlying data

European Nucleotide Archive: Dolichovespula sylvestris (tree wasp). Accession number PRJEB46852; https://identifiers.org/ena.embl/PRJEB46852.

The genome sequence is released openly for reuse. The *D. sylvestris* genome sequencing initiative is part of the Darwin Tree of Life (DToL) project. All raw sequence data and the assembly have been deposited in INSDC databases. The genome will be annotated using the RNA-Seq data and presented through the Ensembl pipeline at the European Bioinformatics Institute. Raw data and assembly accession identifiers are reported in Table 1.

## Author information

Members of the University of Oxford and Wytham Woods Genome Acquisition Lab are listed here: https://doi.org/10.5281/zenodo.5746938.

Members of the Darwin Tree of Life Barcoding collective are listed here: https://doi.org/10.5281/zenodo.5744972.

Members of the Wellcome Sanger Institute Tree of Life programme are listed here: https://doi.org/10.5281/zenodo.6125027.

Members of Wellcome Sanger Institute Scientific Operations: DNA Pipelines collective are listed here: https://doi.org/10.5281/zenodo.5746904.

Members of the Tree of Life Core Informatics collective are listed here: https://doi.org/10.5281/zenodo.6125046.

Members of the Darwin Tree of Life Consortium are listed here: https://doi.org/10.5281/zenodo.5638618.
