# Supplementary material for: The genome sequence of the tree wasp, Dolichovespula sylvestris Scopoli, 1763
Source: Wellcome Open Res. 2022 Mar 28;7:113. [Version 1] doi: 10.12688/wellcomeopenres.17783.1 (PMC9672531; doi:10.12688/wellcomeopenres.17783.1)
Supplement: Peer review history

# Peer review: Reviewer response for version 1

**DOI:** 10.21956/wellcomeopenres.19682.r53341

**Published:** 2022-11-17

**Authors:** Xinhai Ye

**Affiliations:** 1 Zhejiang University, Zhejiang, China

This genome report presents a chromosome-level genome assembly of the tree wasp, *Dolichovespula sylvestris*, which is a eusocial wasp in the family Vespidae. The quality of this genome is very high, with a scaffold N50 of 9.6 Mb and 96% BUSCO score. This genomic resource will facilitate comparative genomics studies of insects in the future, especially in the research field of social insects. The manuscript is well-written and the expression is clear. I only have two minor comments:

1. The authors used the SALSA algorithm for Hi-C scaffolding - this method does not require a specified chromosome number, therefore, I'm wondering if there's any other evidence (such as chromosomic staining) of the 26 scaffolded chromosomes in this study.
2. A single male, not female, wasp was used for genome sequencing. I believe that all experts on Hymenoptera know the reason for the authors' choice; however, I'm not sure if the researchers in other fields know the reason as well. Thus, if possible, I suggest a one-sentence note in the Methods.

Are sufficient details of methods and materials provided to allow replication by others?

Yes

Is the rationale for creating the dataset(s) clearly described?

Yes

Are the datasets clearly presented in a useable and accessible format?

Yes

Are the protocols appropriate and is the work technically sound?

Yes

Reviewer Expertise:

Hymenoptera genomics

I confirm that I have read this submission and believe that I have an appropriate level of expertise to confirm that it is of an acceptable scientific standard.

# Peer review: Reviewer response for version 1

**DOI:** 10.21956/wellcomeopenres.19682.r50702

**Published:** 2022-06-07

**Authors:** Xu Wang

**Affiliations:** 1 Department of Pathobiology, College of Veterinary Medicine, Auburn University, Auburn, AL, USA; 2 HudsonAlpha Institute for Biotechnology, Huntsville, AL, USA

In this manuscript, Falk, Broad, and the Darwin Tree of Life Consortium sequenced and assembled the tree wasp ( *Dolichovespula sylvestris*) genome. The authors combined PacBio long-read data, 10x Genomics linked-read data, as well as Hi-C data to achieve super high quality and continuity of the tree wasp genome. This assembly represents the first Vespinae genome and is an excellent genome resource for comparative genomics and evolution in Hymenopterans.

 Please find specific comments below:

1. Please add BUSCO 5.2.2 to Table 3. Software tools used.
2. “10X” should be “10x”.
3. “Qiagen Plant MagAttract HMW DNA extraction kit” – I was not aware of a plant-specific version of the MagAttract HMW DNA extraction kit (cat no 67563). Could it be a typo? If not, please justify the use of a plant kit for an insect sample.
4. The 26 pseudochromosomes provide valuable information on hymenopteran chromosome evolution. Could the authors provide some information about the karyotype of the tree wasp? How many chromosomes are there for a haploid male genome?
5. The mitochondrial genome assembly accession number is missing. Please provide in the main text or the data table.

Are sufficient details of methods and materials provided to allow replication by others?

Partly

Is the rationale for creating the dataset(s) clearly described?

Yes

Are the datasets clearly presented in a useable and accessible format?

Yes

Are the protocols appropriate and is the work technically sound?

Yes

Reviewer Expertise:

Insect evolution, parasitoid wasps, genomics

I confirm that I have read this submission and believe that I have an appropriate level of expertise to confirm that it is of an acceptable scientific standard.
